# One Health transmission of plasmid-mediated antimicrobial resistance: genomic insights at the interface of food, animals, humans and the environment

**DOI:** 10.1093/jacamr/dlag130

**Published:** 2026-07-04

**Authors:** Daniel F M Monte, Siddhartha Thakur

**Affiliations:** Department of Population Health and Pathobiology, North Carolina State University, College of Veterinary Medicine, Raleigh, NC, USA; Department of Population Health and Pathobiology, North Carolina State University, College of Veterinary Medicine, Raleigh, NC, USA

## Abstract

Antimicrobial resistance (AMR) is increasingly recognized as a complex issue that requires an interdisciplinary One Health approach to find solutions. While early surveillance efforts have emphasized clonal expansion of resistant pathogens, recent genomic studies demonstrate that plasmid-mediated horizontal gene transfer is a dominant force shaping the global AMR landscape. Mobile resistance determinants conferring reduced susceptibility to critically important antimicrobials, including extended-spectrum β-lactams, quinolones, colistin, tigecycline and carbapenems, are now widely detected across food-producing animals, retail foods, environmental waters and human clinical isolates. In this review, we synthesize genomic evidence supporting cross-sector transmission of plasmid-mediated AMR, with a focus on key resistance genes (*bla*_CTX-M_, *qnr*, *mcr*, *tet(X)*, *bla*_NDM_), and their associated plasmid backbones. We discuss why certain plasmids are particularly successful across diverse ecological niches and highlight implications for surveillance and mitigation strategies within a One Health framework. Rather than proposing a new One Health framework, this review synthesizes current genomic evidence highlighting the role of plasmids as major vehicles of AMR dissemination across interconnected reservoirs.

## Introduction

Antimicrobial resistance (AMR) is an ancient and evolving biological phenomenon, shaped by both natural selection and anthropogenic antimicrobial use,^1^ which became one of the most serious threats to modern medicine, compromising the treatment of bacterial infections in humans and animals alike.^2^ The rapid emergence and global dissemination of resistant bacteria are increasingly understood as consequences of interconnected ecological systems linking human populations, food-producing animals, food chains and the environment. This interdependence underpins the importance of the One Health framework, which has become central to international AMR policy and surveillance strategies.^3^

Traditional AMR surveillance has focused predominantly on hospital-associated pathogens and the clonal expansion of resistant lineages. However, whole-genome sequencing (WGS) has revealed that horizontal gene transfer mediated by mobile genetic elements, particularly plasmids, plays a critical role in disseminating resistance across bacterial species and ecological compartments.^4,5^ This shift in understanding has profound implications for risk assessment, as resistance genes can circulate independently of specific bacterial clones.

Of particular concern is the global spread of plasmid-mediated resistance to critically important antimicrobials, including third-generation cephalosporins, quinolone, colistin, tigecycline and carbapenems.^3^ Genes such as *bla*_CTX-M_, *qnr*, *mcr*, *tet(X)* and *bla*_NDM_ are increasingly detected in non-clinical reservoirs, challenging the long-held assumption that high-level resistance emerges primarily in healthcare settings.^5,6^

Several comprehensive reviews have previously addressed plasmid biology, mobile genetic elements and specific resistance determinants such as *mcr* genes.^4,5,7^ However, recent advances in WGS, plasmid reconstruction and One Health surveillance have generated substantial new evidence regarding the dissemination of high-risk resistance plasmids across interconnected human, animal, food and environmental reservoirs. This review aims to synthesize these recent genomic findings within a unified One Health framework, with particular emphasis on plasmid-mediated transmission pathways and their implications for integrated surveillance and control strategies.

## Key plasmid-mediated AMR determinants in a One Health context

### Extended-spectrum β-lactamases at the food–animal–human interface

Extended-spectrum β-lactamase (ESBL)-producing Enterobacterales are a prime example of plasmid-mediated AMR dissemination. Among ESBLs, *bla*_CTX-M_ genes have become globally dominant, although individual variants show distinct ecological associations.^8^ While *bla*_CTX-M-15_ predominates in human clinical *Escherichia coli* lineages, *bla*_CTX-M-55_ and *bla*_CTX-M-65_ are increasingly associated with food-producing animals and foodborne pathogens, particularly *Salmonella enterica*, reflecting the expansion of distinct ESBL lineages within agricultural systems.^8–10^

Genomic investigations have demonstrated that *bla*_CTX-M-65_ is frequently carried by IncFIB(pESI)-like megaplasmids in *Salmonella* Infantis, a globally distributed poultry-associated lineage that is frequently linked to pESI-like megaplasmids carrying AMR and virulence determinants.^11–15^ These plasmids typically encode multiple resistance determinants, virulence-associated genes and toxin–antitoxin systems, enhancing their stability and persistence. Comparative genomic analyses have revealed close relatedness between poultry-derived and human-associated *S*. Infantis isolates, consistent with foodborne transmission pathways and the dissemination of resistant lineages across the food production continuum.^11–15^

Notably, pESI-like plasmids persist over extended periods despite reductions in antimicrobial use, suggesting that plasmid-host co-adaptation and fitness compensation are key contributors to their success.^4^ These findings suggest that antimicrobial stewardship alone may be insufficient to fully mitigate the persistence and dissemination of resistance plasmids across interconnected One Health reservoirs.^4^ The expansion of ESBL-producing *Salmonella* lineages in poultry has direct public health relevance, as poultry remains a major attributed source of human salmonellosis in Europe and elsewhere.^16^

An additional example highlighting the importance of megaplasmids in One Health AMR dynamics is the emergence of pESM-like megaplasmids in *Salmonella enterica* serovar Minnesota.^17^ These large IncFIB-associated plasmids share structural and functional similarities with the pESI-like megaplasmids described in *S*. Infantis, including extensive mosaic architectures, multiple AMR genes, virulence-associated regions and plasmid stability systems.^17^ Genomic studies have shown that pESM-like megaplasmids contribute to the successful persistence and dissemination of multidrug-resistant *S*. Minnesota in poultry production systems, particularly in South America, with increasing evidence of international spread through the food chain.^17^ The convergence of ESBL genes, heavy metal tolerance determinants and maintenance systems on pESM-like megaplasmids underscores their role as high-risk One Health platforms capable of long-term maintenance and cross-sector transmission, reinforcing the need to monitor megaplasmid lineages alongside bacterial clones.

### Colistin resistance and the global spread of *mcr* genes

The discovery of plasmid-mediated colistin resistance encoded by *mcr-1* in *Escherichia coli* and *Klebsiella pneumoniae* represented a watershed moment in AMR research, as it demonstrated for the first time that resistance to a last-resort antimicrobial could disseminate rapidly through horizontal gene transfer.^18^ Since that initial report, a growing family of *mcr* variants (*mcr-1* to *mcr-10*) has been described in Enterobacterales isolated from humans,^19^ food-producing animals,^20^ retail foods^21^ and environmental sources across all inhabited continents.^7^ This rapid diversification underscores the strong selective pressures acting on colistin resistance determinants within interconnected One Health reservoirs.

**Fig. 1.**
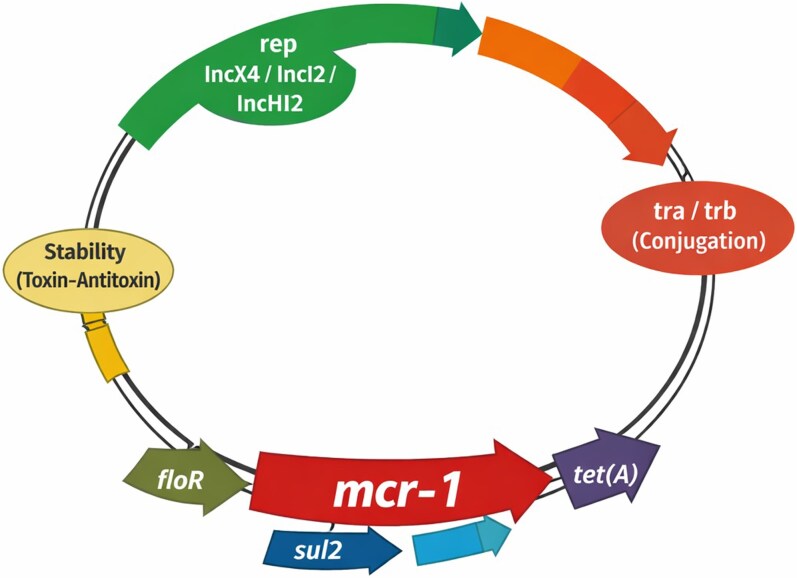
Schematic representation of a **conjugative plasmid** carrying the mcr gene.

Food-producing animals, particularly poultry and swine, have been consistently identified as major reservoirs of *mcr*-harbouring bacteria, reflecting the extensive use of polymyxins in animal production and the subsequent dissemination of resistance determinants across One Health compartments.^7,18,20^ Genomic analyses reveal that *mcr* genes are most frequently located on highly transmissible plasmids belonging to the IncX4, IncI2 and IncHI2 incompatibility groups, which exhibit broad host ranges and high conjugation efficiencies.^7^ These plasmids often display conserved backbones with limited structural variation, facilitating their global dissemination across bacterial species and ecological niches as illustrated in Figure 1. The detection of nearly identical *mcr*-bearing plasmids in animal, food and human isolates provides compelling evidence for plasmid-level transmission along the food chain.

Environmental compartments further amplify the dissemination and persistence of *mcr* genes. Surface waters, wastewater treatment plants and agricultural runoff serve as convergence points for bacteria originating from human, animal and wildlife sources, creating ideal conditions for horizontal gene transfer.^6^ WGS studies have demonstrated that environmental isolates carrying *mcr* genes often harbour plasmids closely related to those detected in clinical and agricultural settings, supporting the concept of environmental waters as long-term reservoirs rather than transient sinks.^6^

Importantly, the continued detection of *mcr* genes in regions where colistin use in food animals has been substantially reduced suggests that factors beyond direct antimicrobial exposure contribute to their maintenance. Co-selection driven by the linkage of *mcr* genes with resistance determinants to other antimicrobials, as well as genes conferring tolerance to heavy metals and biocides, appears to play a significant role in sustaining these plasmids within microbial communities.^5^ In addition, plasmid stability systems and compensatory adaptations may reduce the fitness costs associated with *mcr* carriage, facilitating long-term persistence even under reduced selective pressure.

Together, these findings indicate that *mcr* genes have become entrenched within different ecosystems, complicating efforts to reverse colistin resistance through stewardship alone. Effective mitigation will require integrated surveillance across human, animal and environmental sectors, with particular emphasis on tracking high-risk plasmid lineages and identifying ecological drivers that promote their persistence and spread.

### Tigecycline resistance and the emergence of *tet(X)*

Tigecycline is widely regarded as a last-line antimicrobial for the treatment of infections caused by multidrug-resistant Gram-negative bacteria, particularly those resistant to carbapenems and third-generation cephalosporins. The emergence of plasmid-mediated tigecycline resistance encoded by *tet(X)* variants therefore represents a critical threat to antimicrobial chemotherapy, as it compromises one of the few remaining therapeutic options for severe infections.^22^

*tet(X)* was originally identified in anaerobic bacteria, particularly Bacteroides species, where it encodes a flavin-dependent monooxygenase capable of enzymatically inactivating tetracyclines, including tigecycline.^23^ However, its clinical significance remained limited until the subsequent identification of mobile *tet(X)* variants in *Acinetobacter* species and Enterobacterales, which revealed the potential for horizontal dissemination of tigecycline resistance.^22,24^ The subsequent discovery of mobile *tet(X3)* and *tet(X4)* variants marked a paradigm shift, revealing that tigecycline resistance could disseminate efficiently through horizontal gene transfer.^22,24^ These variants have now been reported in *Escherichia coli*, *Salmonella enterica* and other Enterobacterales isolated from food-producing animals, retail meat products and environmental samples across multiple continents, indicating widespread circulation beyond clinical settings.^22,24,25^

Genomic analyses show that *tet(X)* genes are frequently embedded within conjugative plasmids and complex resistance regions that facilitate interspecies transfer. These plasmids often possess broad host ranges and harbour additional AMR determinants, including genes conferring resistance to tetracyclines, β-lactams, aminoglycosides and sulphonamides.^22,24^ Such genetic linkage markedly increases the potential for co-selection under diverse selective pressures, even in the absence of tigecycline exposure.

Environmental reservoirs may play an important role in the ecology of *tet(X)*.^22,25^ Most reports of *tet(X)*-carrying bacteria have originated from food-producing animals and animal-associated production environments, suggesting that agricultural settings may contribute to the maintenance and dissemination of these resistance determinants. Such environments create opportunities for interaction among bacteria from animal, human and environmental sources, potentially facilitating horizontal gene transfer.^22,25^ WGS has further demonstrated that *tet(X)*-bearing plasmids recovered from environmental and food-associated isolates can be closely related to those detected in clinical strains, supporting the hypothesis of plasmid-mediated transmission across One Health compartments.^22,24^

The undetected dissemination of *tet(X)* in non-clinical reservoirs is particularly concerning, as tigecycline resistance may become established before being recognized in clinical surveillance systems. Together, these observations underscore the urgent need for proactive One Health surveillance strategies that integrate genomic monitoring of food, animal and environmental reservoirs.^22,25^ Early detection of high-risk *tet(X)*-carrying plasmids will be essential to prevent further erosion of last-resort treatment options and to inform targeted intervention measures.

### Carbapenemases beyond the hospital setting

Carbapenem resistance, driven by diverse carbapenemase enzymes, has traditionally been regarded as a problem confined to healthcare environments;^26^ however, accumulating genomic evidence indicates that plasmid-mediated carbapenemase genes, including *bla*_NDM_, *bla*_KPC_ and *bla*_OXA-48_-like variants, are increasingly detected in food-producing animals, retail foods and environmental waters.^5,27,28^ The global dissemination of carbapenemase-producing Enterobacterales, initially documented in clinical settings, has since expanded beyond hospitals into community, animal and environmental reservoirs.^6,27^ These findings challenge the long-standing paradigm that carbapenem resistance emerges exclusively under clinical selective pressures and instead highlight the role of non-clinical reservoirs in sustaining and disseminating these determinants.

Carbapenemase genes identified outside hospitals are frequently carried on broad-host-range plasmids, particularly those belonging to the IncHI2, IncX and IncA/C incompatibility groups, which are capable of stable maintenance across diverse members of the Enterobacterales.^5,27^ These plasmids often co-harbour additional resistance determinants, including genes conferring resistance to extended-spectrum β-lactams, aminoglycosides and fluoroquinolones, thereby facilitating co-selection in environments where carbapenems are not used. Such genetic linkage may explain the persistence of carbapenemase genes in agricultural and environmental settings despite the absence of direct carbapenem exposure.

Environmental waters impacted by wastewater discharge, livestock effluents and agricultural runoff appear to play a critical role in the ecology of carbapenemase-producing organisms.^6,29^ Rivers, irrigation channels and wastewater treatment plants act as convergence points for bacteria originating from hospitals, communities, farms and wildlife, creating favourable conditions for horizontal gene transfer.^6^ Metagenomic analyses of urban sewage have detected clinically relevant resistance genes, including carbapenemases such as *bla*_NDM_, highlighting the presence of these determinants in environmental reservoirs.^30^

Notably, the detection of *bla*_NDM-_ and *bla*_OXA-48_-like genes in food animals and aquatic environments raises concerns regarding silent dissemination through food chains and water reuse systems. These reservoirs may serve as long-term sources for reintroduction of carbapenem resistance into human populations, undermining infection control efforts focused solely on healthcare settings. Together, these observations underscore the necessity of integrating environmental and food-sector surveillance into carbapenem resistance monitoring frameworks and highlight plasmids as critical targets for One Health–oriented intervention strategies.

Importantly, the epidemiology of carbapenemase genes differs from that of resistance determinants such as *mcr* and *tet(X)*, which are more directly associated with antimicrobial classes historically used in food–animal production. In contrast, carbapenemase-producing bacteria are generally detected at relatively low frequencies in food-producing animals and food products, particularly in regions where carbapenems are not used in animal agriculture. Their occurrence in non-clinical settings is therefore frequently attributed to environmental contamination, wastewater dissemination or spillover from human-associated reservoirs. These differences highlight that distinct resistance determinants may follow different selection pressures and transmission pathways across One Health compartments.^5,27,28^

### Plasmid-mediated fluoroquinolone resistance: the role of *qnr* genes

Fluoroquinolones are critically important antimicrobials widely used in both human and veterinary medicine. While chromosomal mutations in genes encoding DNA gyrase and topoisomerase IV remain the primary mechanism of high-level fluoroquinolone resistance, plasmid-mediated quinolone resistance (PMQR) determinants have emerged as important contributors to reduced susceptibility and the stepwise evolution of resistance. Among these, *qnr* genes represent the most extensively characterized PMQR mechanism.

*qnr* genes encode pentapeptide repeat proteins that protect DNA gyrase and topoisomerase IV from quinolone inhibition, thereby conferring low-level resistance that facilitates the selection of additional chromosomal mutations.^31,32^ Multiple *qnr* variants, including *qnrA*, *qnrB* and *qnrS*, have been widely reported in Enterobacterales isolated from humans, food-producing animals, retail foods and environmental sources, highlighting their broad distribution across One Health compartments.^31^

Genomic analyses indicate that *qnr* genes are frequently located on conjugative plasmids belonging to incompatibility groups such as IncF, IncN, IncHI2 and IncQ, which often co-harbour additional resistance determinants, including extended-spectrum β-lactamase (ESBL) genes and aminoglycoside resistance genes.^5,32^ This co-localization promotes co-selection under diverse antimicrobial pressures, allowing *qnr*-bearing plasmids to persist and disseminate even in the absence of direct fluoroquinolone exposure.

Within food production systems, *qnr*-harbouring plasmids have been detected in *Escherichia coli*^33^ and *Salmonella enterica* isolates from poultry,^34^ swine^35^ and retail meat,^34^ suggesting that food chains represent important pathways for transmission to humans. Comparative genomic studies have demonstrated the presence of highly similar *qnr*-carrying plasmids in animal and human isolates, supporting the role of foodborne transmission in the dissemination of PMQR determinants.^32^

Environmental reservoirs further contribute to the ecology of *qnr* genes. Surface waters, wastewater and agricultural environments serve as interfaces where bacteria from human and animal sources converge, facilitating horizontal gene transfer and the maintenance of PMQR plasmids.^36^ The detection of *qnr*-positive Enterobacterales in aquatic environments underscores the role of environmental compartments as both reservoirs and transmission hubs within One Health systems.^36^

Although *qnr* genes alone typically confer only low-level resistance, their epidemiological significance lies in their ability to promote the selection of higher-level resistance mechanisms and to spread efficiently via mobile genetic elements. Consequently, *qnr*-carrying plasmids represent important intermediates in the evolution of fluoroquinolone resistance and should be considered in integrated genomic surveillance strategies aimed at capturing the full spectrum of AMR dissemination across human, animal and environmental sectors.

The major plasmid types, associated resistance genes and their distribution across One Health reservoirs are summarized in Table 1.

**Table 1.** Major plasmid types associated with key antimicrobial resistance genes across One Health reservoirs

Resistance gene	Plasmid type	Bacterial host	Source (human/animal/environment)	Key features
*bla*_CTX-M-65_	IncFIB (pESI-like)	*Salmonella* Infantis	Poultry, humans	ESBL, virulence, megaplasmid
*mcr-1*	IncX4/IncI2/IncHI2	*E. coli*, *Salmonella*	Animals, food, environment	High transfer rate
*tet(X4)*	IncX1/IncFII	*E. coli*	Animals, environment	Tigecycline resistance
*bla*_NDM_	IncX3/IncFII	Enterobacterales	Humans, environment	Carbapenemase
*qnrS* / *qnrB*	IncF/IncN/IncHI2	*E. coli*, *Salmonella*	Animals, food	PMQR, co-selection

## Genomic evidence for plasmid dissemination at the human, animal, environment interface

### What genomics has changed

WGS has fundamentally transformed AMR epidemiology by providing unprecedented resolution for tracking bacterial populations and the mobile genetic elements that shape their evolution. High-resolution approaches such as core genome multilocus sequence typing and single nucleotide polymorphism-based phylogenetic analysis enable robust discrimination between clonal expansion and horizontal gene transfer, allowing transmission pathways to be inferred with far greater confidence than was previously possible using phenotypic or low-resolution molecular methods.^37,38^ Within a One Health framework, these approaches allow investigators to identify whether resistant bacteria detected in humans, animals, foods and environmental reservoirs are epidemiologically linked, thereby improving our understanding of cross-sector transmission pathways.^37,38^

A critical advance enabled by genomics is the ability to decouple bacterial chromosomal lineages from resistance gene dissemination. Comparative genomic analyses have demonstrated that genetically unrelated bacterial isolates from different hosts, geographic regions and ecological compartments can carry nearly identical resistance plasmids, highlighting plasmids as independent evolutionary entities.^39^ This paradigm shift has profound implications for One Health based surveillance, as it reveals that AMR risk cannot be fully assessed by tracking bacterial species or sequence types alone.

The development of plasmid reconstruction and typing tools has further enhanced understanding of AMR transmission dynamics.^39^  *In silico* approaches, including plasmid multilocus sequence typing, replicon typing and hybrid assembly strategies, allow detailed characterization of plasmid backbones, resistance regions and mobility-associated genes. The integration of long-read sequencing, particularly in hybrid assembly approaches, has been essential for resolving complete plasmid architectures and accurately assigning resistance genes to specific mobile genetic elements, thereby facilitating the reconstruction of plasmid transmission pathways across One Health compartments. These analyses have uncovered conserved plasmid lineages, such as IncFIB, IncHI2 and IncX plasmids, that persist across human, animal, food and environmental reservoirs, often over extended temporal and geographic scales. Consequently, plasmid reconstruction is increasingly recognized as an important component of One Health surveillance because it enables direct tracking of resistance vehicles across otherwise unrelated bacterial populations.^39,40^

Importantly, WGS-based investigations have also enabled the integration of chromosomal phylogenies with plasmid architectures, providing a comprehensive framework for disentangling complex One Health transmission networks. By revealing instances in which plasmid-mediated resistance spreads across sectors in the absence of clonal bacterial transmission, genomics has reshaped our understanding of AMR ecology and underscored the need for surveillance strategies that explicitly target mobile genetic elements alongside bacterial hosts.

These approaches have revealed conserved plasmid lineages and structural backbones that persist across bacterial hosts and ecological compartments. Examples include pESI-like megaplasmids associated with *Salmonella* Infantis and the pESM megaplasmid recently described in *Salmonella* Minnesota, both of which illustrate the long-term persistence and dissemination of successful plasmid backbones within food-associated bacterial populations.^11,17^

### Genomic evidence of plasmid-mediated AMR across human, animal and environmental reservoirs

A growing body of genomic evidence documents the transmission of plasmid-mediated AMR across human, animal and environmental sectors, highlighting the permeability of traditional surveillance boundaries. In poultry production systems, *Salmonella enterica* lineages carrying ESBL-encoding pESI-like megaplasmids have been shown to cluster phylogenetically with human clinical isolates, providing strong support for foodborne transmission pathways.^11^ High-resolution WGS analyses reveal limited chromosomal divergence between poultry- and human-associated isolates, alongside near-identical plasmid backbones, indicating that both clonal expansion and plasmid-mediated horizontal gene transfer contribute to the persistence of these resistant lineages.

Environmental surveillance has further demonstrated that surface waters act as important reservoirs and conduits for AMR. Genomic studies have identified rare and emerging resistance determinants, including ESBLs, *mcr* genes and carbapenemases, in riverine and wastewater-associated Enterobacterales that mirror those found in clinical and agricultural contexts.^6^ In many cases, environmental isolates harbour plasmids with high sequence similarity to those recovered from human and animal sources, supporting the hypothesis that environmental waters facilitate plasmid-level dissemination across One Health compartments rather than serving merely as passive sinks.

Backyard and small-scale poultry systems represent particularly underappreciated interfaces where humans, animals and the environment interact closely, often in the absence of formal biosecurity measures. Genomic investigations in the United States have demonstrated a high prevalence and diversity of ESBL-producing *Escherichia coli* in backyard poultry production systems, frequently carrying plasmid-mediated *bla*_CTX-M_ genes, highlighting the potential for dissemination of resistance determinants at the human–animal interface.^41^

Beyond backyard settings, retail meat surveillance studies further support the role of food systems in the dissemination of AMR. Analyses of *E*. *coli* from retail meat products in North Carolina revealed diverse resistance profiles and the presence of clinically relevant resistance determinants, underscoring the continuity between food production systems and human exposure pathways.^42^

In addition to direct animal and food contact, the integration of animal production systems with environmental compartments creates further opportunities for dissemination. Environmental contamination associated with poultry production can contribute to the spread of antimicrobial-resistant bacteria and plasmids into surrounding matrices, including soil, water and fresh produce. These findings suggest that fresh produce may act as an additional vehicle for the transmission of plasmid-mediated resistance, linking agricultural environments with human exposure pathways.

Collectively, these observations indicate that backyard poultry, retail food systems and associated agro-environmental interfaces function as critical nodes in One Health AMR ecology, facilitating the introduction, persistence and dissemination of high-risk plasmids across human, animal and environmental compartments.

Genomic investigations in these settings consistently reveal high diversity of ESBL-producing *Escherichia coli* and other Enterobacterales, frequently carrying plasmids closely related to those detected in commercial poultry production and human clinical isolates.^5^ These findings suggest that backyard poultry may function as bidirectional bridges, enabling both the introduction of resistant bacteria from industrial food systems into households and the dissemination of resistance determinants into the surrounding environment.

Collectively, these cross-sector examples illustrate that plasmid-mediated AMR dissemination operates across interconnected ecological networks rather than within isolated reservoirs. The convergence of similar resistance plasmids in humans, food animals and environmental matrices underscores the necessity of integrated One Health surveillance approaches capable of capturing plasmid flow across sectors and identifying critical control points for intervention.

### Why plasmids succeed across One Health compartments

The success of plasmids across diverse One Health compartments reflects a convergence of genetic, ecological and evolutionary factors that promote their stability, transmissibility and long-term persistence. Many high-risk plasmids encode sophisticated maintenance systems, including toxin–antitoxin modules, partitioning functions and addiction mechanisms, which ensure faithful inheritance during bacterial cell division and reduce the likelihood of plasmid loss in the absence of selective pressure.^4^ These features effectively transform plasmids into stable genetic elements that behave as semi-autonomous units of selection within bacterial populations.

High-risk plasmid groups repeatedly identified throughout this review, including IncFIB(pESI-like), IncHI2, IncX4 and IncX3, exemplify these adaptive characteristics. Their success is often attributed to a combination of efficient conjugative transfer, broad host range, stable maintenance systems and the frequent co-localization of AMR, virulence and stress tolerance determinants. These features enhance persistence across diverse ecological compartments and increase opportunities for dissemination through interconnected One Health networks.^4,5,15^

Beyond intrinsic stability mechanisms, resistance genes carried on plasmids are frequently embedded within complex genetic regions that include determinants conferring tolerance to heavy metals, disinfectants and biocides. Such co-localization facilitates co-selection in agricultural, aquaculture and environmental settings where exposure to metals, disinfectants or other stressors is common, even when antibiotic use is limited.^6^ As a result, plasmids encoding AMR can be maintained and disseminated under a wide range of non-antibiotic selective pressures.

Ecological drivers further reinforce plasmid persistence across One Health systems. Antimicrobial use in human medicine and food production, inadequate sanitation infrastructure and environmental contamination with pharmaceuticals and industrial compounds create heterogeneous selective landscapes that favour bacteria harbouring mobile resistance elements. In environments such as farms, food-processing facilities and surface waters, repeated opportunities for bacterial contact facilitate horizontal gene transfer, amplifying plasmid spread across species and ecological boundaries.

From an evolutionary perspective, compensatory adaptations play a critical role in plasmid success. Initially, plasmid carriage may impose fitness costs on bacterial hosts; however, compensatory mutations in either the plasmid or the chromosome can mitigate these costs over time, allowing plasmids to persist even when selective pressure is reduced or absent.^5^ This evolutionary stabilization helps explain the long-term maintenance of high-risk plasmids, such as ESBL- and *mcr*-carrying elements, across human, animal and environmental reservoirs.

Collectively, these mechanisms illustrate why plasmids are exceptionally well suited to exploit interconnected One Health ecosystems. Their ability to persist, adapt and disseminate across diverse hosts and environments positions them as central drivers of AMR emergence, underscoring the need for surveillance and intervention strategies that explicitly target plasmid dynamics rather than bacterial lineages alone.

## Implications for surveillance and control

Recognition of plasmids as central drivers of AMR dissemination necessitates a fundamental re-evaluation of current surveillance and control strategies. Many existing monitoring programmes remain focused on specific bacterial species, resistance phenotypes or clinical isolates, which may fail to capture the broader dynamics of plasmid-mediated transmission occurring across food, animal and environmental reservoirs.^43^ As a result, emerging resistance determinants may circulate undetected for prolonged periods before being recognized in clinical settings.

Integrated One Health surveillance frameworks that incorporate WGS data from human, veterinary, food and environmental sources are essential for the early detection of high-risk resistance genes and plasmid lineages. Genomic approaches that explicitly track plasmid backbones, replicon types and resistance gene cassettes can reveal transmission pathways that are invisible to species-centric surveillance. Harmonization of sequencing protocols, metadata standards and analytical pipelines across sectors will be critical to enable meaningful cross-sector comparisons and data integration.

From a control perspective, targeting plasmids rather than bacterial lineages offers a complementary and potentially more effective framework for AMR mitigation. Interventions aimed at reducing the selective pressures that promote plasmid persistence, such as unnecessary antimicrobial use, heavy metal exposure and biocide overuse may have broader impacts than strategies focused on individual pathogens. In food production systems, improved biosecurity, waste management and environmental controls can reduce opportunities for horizontal gene transfer and plasmid dissemination.

Emerging analytical approaches further expand the potential of genomic surveillance. Predictive genomics and machine-learning models can integrate genomic, ecological and epidemiological data to forecast AMR emergence and identify hotspots of plasmid transmission. Such tools may support risk-based surveillance and guide targeted interventions at critical points along the human–animal–environment interface. Ultimately, effective control of plasmid-mediated AMR will require coordinated, interdisciplinary strategies that bridge microbiology, genomics, environmental science and public health policy within a unified One Health framework.

Despite the growing body of evidence supporting the role of plasmids in AMR dissemination, several important limitations should be acknowledged. First, the relative contributions of plasmid-mediated horizontal gene transfer and clonal expansion likely vary among bacterial species, resistance determinants, geographic regions and production systems.^5^ For example, while *bla*_CTX-M_ genes are frequently associated with transmissible plasmids, chromosomal integration and the expansion of successful resistant lineages have also contributed substantially to the global epidemiology of ESBL-producing Enterobacterales.^5,8^ Second, although recent studies have identified backyard poultry systems as potential interfaces for the exchange of resistance determinants between humans, animals and the environment, the extent to which these findings can be generalized across different production systems and geographic regions remains uncertain.^41^ Finally, environmental surveillance studies increasingly detect resistance genes and plasmids in surface waters, wastewater and agricultural environments; however, the ecological significance of these findings is not always clear.^6,29^ Detection of resistance determinants does not necessarily demonstrate long-term persistence, active plasmid transfer or sustained transmission within environmental bacterial communities. Future studies integrating longitudinal sampling, culture-based approaches, long-read sequencing and transmission analyses will be essential to better define the relative importance of these mechanisms and reservoirs in One Health AMR dissemination.

Several emerging developments are likely to further transform the study of plasmid-mediated AMR within a One Health framework. Advances in long-read sequencing technologies are increasingly enabling complete reconstruction of resistance plasmids and their transmission pathways across ecological compartments. In parallel, metagenomic approaches are expanding the ability to characterize resistomes directly from complex environmental and animal-associated samples without prior culture. Furthermore, the integration of large-scale genomic datasets with machine learning and predictive modelling may improve the early detection of high-risk plasmids and support proactive intervention strategies. Together, these developments are expected to enhance the resolution and effectiveness of integrated One Health surveillance systems.

### Conclusions

Plasmid-mediated AMR represents a unifying threat across One Health compartments, undermining the effectiveness of critically important antimicrobials. Genomic evidence increasingly demonstrates that food and environmental reservoirs actively sustain and disseminate resistance genes once thought to be confined to clinical settings.

Addressing this challenge will require harmonized surveillance, interdisciplinary research and coordinated policy interventions extending beyond healthcare. Integrating genomic insights with One Health principles offers a path towards disrupting key transmission pathways and slowing the global spread of AMR.
